# *Granzyme G *is expressed in the two-cell stage mouse embryo and is required for the maternal-zygotic transition

**DOI:** 10.1186/1471-213X-10-88

**Published:** 2010-08-12

**Authors:** Tung-Chou Tsai, William Lin, Shang-Hsun Yang, Winston TK Cheng, En-Hui Cheng, Maw-Sheng Lee, Kowit-Yu Chong, Chuan-Mu Chen

**Affiliations:** 1 Department of Life Sciences, National Chung Hsing University, Taichung 402, Taiwan; 2 Department of Physiology, National Cheng Kung University, Tainan 701, Taiwan; 3 Department of Animal Science and Biotechnology, Tunghai University, Taichung 407, Taiwan; 4 Division of Infertility Clinic, Lee Women's Hospital, and Chung Shan Medical University, Taichung 406, Taiwan; 5 Department of Medical Biotechnology and Laboratory Science, Chang Gung University, Tao-Yuan 333, Taiwan

## Abstract

**Background:**

Detailed knowledge of the molecular and cellular mechanisms that direct spatial and temporal gene expression in pre-implantation embryos is critical for understanding the control of the maternal-zygotic transition and cell differentiation in early embryonic development. In this study, twenty-three clones, expressed at different stages of early mouse development, were identified using differential display reverse transcription polymerase chain reaction (DDRT-PCR). One of these clones, which is expressed in 2-cell stage embryos at 48 hr post-hCG injection, shows a perfect sequence homology to the gene encoding the granzyme G protein. The granzyme family members are serine proteases that are present in the secretory granules of cytolytic T lymphocytes. However, the pattern of granzyme G expression and its function in early mouse embryos are entirely unknown.

**Results:**

Upon the introduction of an antisense morpholino (2 mM) against granzyme G to knock-down endogenous gene function, all embryos were arrested at the 2- to 4-cell stages of egg cleavage, and the *de novo *synthesis of zygotic RNAs was decreased. The embryonic survival rate was dramatically decreased at the late 2-cell stage when serine protease-specific inhibitors, 0.1 mM 3,4-dichloroisocoumarin (3,4-DCI), and 2 mM phenyl methanesulphonyl fluoride (PMSF), were added to the *in vitro *embryonic culture medium. Survival was not affected by the addition of 0.5 mM EDTA, a metalloproteinase inhibitor.

**Conclusion:**

We characterized for the first time the expression and function of *granzyme G *during early stage embryogenesis. Our data suggest that granzyme G is an important factor in early mouse embryonic development and may play a novel role in the elimination of maternal proteins and the triggering of zygotic gene expression during the maternal-zygotic transition.

## Background

Mammalian embryonic development at pre-implantation stages involves rapid cell proliferation and the earliest phases of cell differentiation. Fertilization triggers the completion of meiotic division in the oocyte, induces embryonic processes such as the degradation of maternal RNAs and proteins, and activates the embryonic genome for the maternal-zygotic transition (MZT). It has been proposed that the activation of the embryonic genome begins at the 2-cell stage in mouse embryos, the 4- to 8-cell stage in human embryos, and the 8- to 16-cell stage in rabbit and sheep embryos [1]. Early genes such as *Zar1 *[2], *ezrin *[3], *hsp70.1 *[4], and *U2afbp-rs *[5] may play important roles in embryonic genome activation. Previous work has suggested that the acquisition of a transcriptionally repressive environment and changes in the chromatin structure caused by alterations in histone deacetylase activity can block or stimulate the repression of markers of genome activation [6,7]. However, the transition of the control from the maternal to the embryonic genome in early mammalian embryos is still not fully understood.

As a first step towards the elucidation of factors important for the proper functioning of early mouse embryonic development, we used the differential display reverse transcription polymerase chain reaction (DDRT-PCR) method [8,9] to compare two or more mRNA samples prepared from small amounts of tissue. This method is particularly suitable for developmental studies that involve temporal changes in gene expression in pre-implantation embryos. Genes that are temporally and differentially expressed in mouse embryos have been identified using this technique [10,11]. In the current study, tweenty-three mRNA molecules that are differentially expressed in unfertilized eggs, 2-cell, or 4-cell embryos were detected. One of these clones, expressed in 2-cell stage embryos at 48 hr post-hCG injection, has perfect sequence homology with the gene encoding granzyme G.

The expression of members of the granzyme gene family of proteins (granzymes A-H, K, M), which encode serine proteases, has been documented in the secretory granules of cytolytic T lymphocyte lines [12]. Granzymes D, E, F, and G have also been shown to be expressed at gestation in the mouse uterus during the process of decidualization, in which rapid uterine cell growth and differentiation occurs [13]. The decidual reaction is primarily characterized by the differentiation of stromal fibroblasts into decidual cells and by the proliferation and differentiation of the granulated metrial gland (GMG) cells [14]. Murine GMG cells belong to the natural killer (NK) cell lineage [15-17], and an analogous cell type, the endometrial granulocyte, has been identified in humans [18]. In the mouse, GMG cell differentiation begins at about day 7 of gestation and manifests via the accumulation of cytolytic mediators, including perforin and granzymes A-H, within cytoplasmic granules. GMG cells have been proposed to regulate trophoblast invasion into maternal deciduas. Indeed, trophoblast killing by murine and human uterine NK cells has been reported [19,20]. Nevertheless, the expression of *granzyme G *and its function in early embryos are entirely unknown.

In this study, the function of granzyme G during early embryonic development was elucidated using morpholino oligonucleotides to knock-down *granzyme G*-specific mRNA translation and granzyme-specific serine protease inhibitors to inhibit protein activity in an *in vitro *culture system. The embryo survival rate, cleavage rate, 2-cell developmental block effect, and inner cell mass (ICM) morphology were evaluated extensively.

## Results

### Identification of differentially expressed genes in mouse embryos during early developmental stages

**Figure 1:**
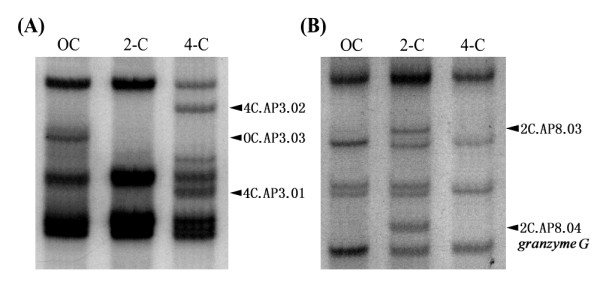
**Representative profiles of differential display mRNAs at various stages of mouse development**. Polymerase chain reaction amplicons were amplified using the primer sets of **(A) **random primer H-AP3 and anchor primer T11G, and **(B) **random primer H-AP8 and anchor primer T11G. Mouse embryos at the unfertilized oocyte (OC), two-cell embryo (2-C), and four-cell embryo (4-C) stages were used in this study and are shown at the top of each lane. Differentially displayed bands are marked by arrows.

For DDRT-PCR, ten different random primers and four anchor primers were used in 40 unique combinations. For templates, total RNA was prepared from various stages of developing embryos post-hCG injection. Thirty-one of the forty random-anchor primer combinations produced DDRT-PCR patterns that were identical, irrespective of the developmental stage of the mouse embryos (data not shown). Only nine random-anchor primer combinations detected transcripts that were clearly differentially expressed. Representative DDRT-PCR patterns are shown in Figure 1. Transcripts that were clearly differentially expressed at a particular egg cleavage stage were identified for further analysis. A total of tweenty-three such bands were isolated, including six cDNA clones in unfertilized eggs, twelve cDNA clones in 2-cell embryos, and five cDNA clones in 4-cell embryos (Table 1). The size of these bands ranged from 250 to 870 bp.

**Table 1:** Differential display mRNAs identified from early mouse embryos by DDRT-PCR analysis

Stage	dbEST ID	**GenBank AccN**^**†**^	Description
Oocyte	7302701	BF733166	AC133163.2: Mus musculus chromosome 9 clone RP24-338G17.
	7302702	BF733167	NT_039260.7: Mus musculus chromosome 4 genomic contig, tyrosinase-related protein 1
	7302703	BF733168	NT_166285.1: Mus musculus chromosome 3 genomic contig, 226046 bp at 5' side: hypothetical protein
	7302704	BF733169	NT_039424.7: Features in this part of subject sequence: ubiquitin protein ligase E3A isoform 2
	7302706	BF733171	NM_001001176.1: Mus musculus TATA box binding protein (TBP)-associated factor 9 (Taf9)
2-Cell	6920934	BF440092	AL845157.7: Ortholog of H. sapiens chromosome 2open reading frame 25 (C2orf25) and part of a novel gene
	6920935	BF440093	AG509034: Mus musculus molossinus DNA, clone: MSMg01- 412D07.T7, genomic survey sequence
	6920936	BF440094	AL670999: Contains the (pseudo) gene for a novel protein similar to high-mobility group box 1 (Hmgb1)
	6920937	BF440095	NM_001001176: Mus musculus TAF9B RNA polymerase II
	6920938	BF440096	AC109179: Mus musculus chro. 18 from clone RP23-382N7
	6920939	BF440097	NT_039240.7: Ndst4; N-deacetylase/N-sulfotransferase (heparinglucosaminyl) 4
	6920940	BF440098	AC206551.4: Pongo abelii BAC clone CH276-44A10 from chromosome 15, complete sequence
	6920941	BF440099	NM_025948.2: Mus musculus LSM14 homolog A (SCD6,S.cerevisiae)
	6920942	BF440100	BC054388: Mus musculus ribosomal protein L37
	6920943	BF440101	AC068254.6: Homo sapiens chromosome 18, clone RP11-543H23, complete sequence.
	6920944	BF440102	BT043574.1: Salmo salar clone HM4_2567 membrane protein palmitoylated 1 mRNA, complete cds.
	7302695	BF733160	J02872: Mouse granzyme G mRNA
4-Cell	7302696	BF733161	NT_039471.7: Mus musculus chromosome 9 genomic contig, strain C57BL/6J
	7302697	BF733162	AF375046: Mus musculus ATP-dependent chromatin remodeling protein SNF2 H mRNA
	7302698	BF733163	AK076019: Mus musculus Methylenetetrahydrofolate dehydrogenase (Mthfd1)
	7302699	BF733164	NM_001001984.2: Mus musculus lysine (K)-specific demethylase 2A
	7302700	BF733165	AC198183.2: Nomascus leucogenys BAC clone CH271-340F4 from chromosome unknown, complete sequence

^† ^All of the new identified mouse embryonic cDNA sequences were deposited in GenBank.

### Purification and sequencing of cDNAs from differential display amplicons

To further characterize the differentially expressed embryonic transcripts, the bands were excised from the gel for PCR re-amplification using the same primer combinations that had led to their initial identification. In all cases, PCR re-amplification was successful, and the size of the subsequent PCR products matched those of the original DDRT-PCR products. The PCR amplicons were cloned and subjected to sequence analysis. In a comprehensive search of the GenBank database, the sequences of eight of the cDNA clones matched perfectly with known mouse genes: *granzyme G*, methylenetetrahydrofolate dehydrogenase (*Mthfd1*), *TAF9B *RNA polymerase II, LSM14 homlog A (*SCD6*), N-deacetylase/N-sulfotransferase 4 (*Ndst 4*), the ATP-dependent chromatin remodeling protein *SNF2H*, high-mobility group box 1 (*Hmgb1*), and the TATA-box binding protein-associated factor (*Taf9*) (Table 1). Another group of 15 cDNA clones showed high sequence homology (60-87%) with existing mouse genes, and may represent novel members of these gene families. As the *granzyme G *gene is known to encode a serine protease that is present in the secretory granules of cytolytic T lymphocyte lines and granzymes D-G are also expressed during late gestation in the mouse uterus, we thought it would be worthwhile to investigate whether granzyme G had a novel, specific function in early embryonic development.

### Granzyme G gene expression profiling at different mouse developmental stages

**Figure 2:**
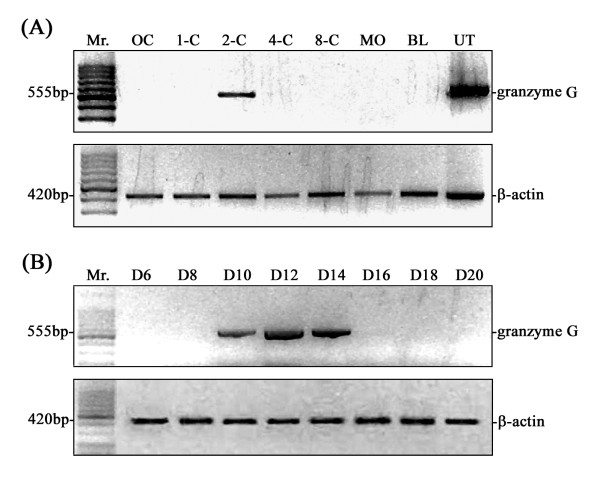
**Temporal and spatial expression of granzyme G mRNA during early embryonic development**. **(A) **RT-PCR confirmation of the temporally expressed granzyme G gene identified by DDRT-PCR in 2-cell stage embryos. Mouse embryos were collected at unfertilized oocyte (OC), one-cell (1-C), two-cell (2-C), four-cell (4-C), eight-cell (8-cell), morula (MO), and blastocyst (BL) stages. Pregnant mouse uterine (UT) GMG cell mRNA was used as a positive control. (B) Stage-specific expression of granzyme G in the extraembryonic trophoblast during early implantation (D6-D8), middle implantation (D10-14), and late implantation (D16-20). A β-actin primer set was used as an internal control. The results are representative of three experiments.

**Figure 3:**
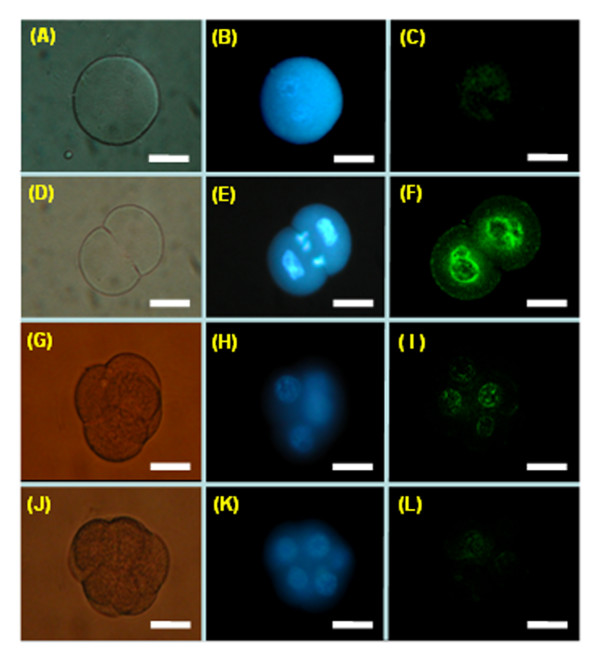
**Whole-mount embryo in situ hybridization (ISH) using a FITC-conjugated *granzyme G *oligonucleotide probe at different stages of normal mouse development**. **(A to C) **One-cell stage mouse embryos. **(D to F) **Two-cell stage mouse embryos. **(G to I) **Four-cell stage mouse embryos. **(J to K) **Eight-cell stage mouse embryos. The left panels (A, D, G, and J) show embryos photographed under phase contrast imaging. The middle panels (B, E, H, and K) show embryos stained with Hoechst 33342 for DNA localization (blue) under fluorescence microscope observation. The right panels (C, F, I, and L) show embryos hybridized with a *granzyme G *oligonucleotide probe (green) under confocal microscopic observation. Scale bar: 30 μm.

**Figure 4:**
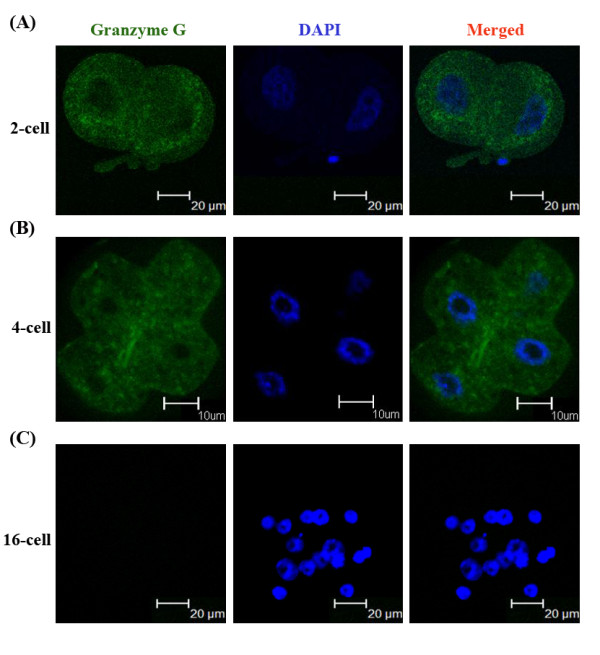
**Immunoflourescence staining of Granzyme G protein expression and subcellular localization at different stage embryos**. **(A) **Two-cell stage mouse embryos (n = 6). **(B) **Four-cell stage mouse embryos (n = 6). **(C) **Sixteen-cell stage mouse embryos (n = 5). The left panels show embryos stained with a granzyme G-specific primary antibody and FITC-conjugated secondary IgG antibody. The middle panels show embryos stained with DAPI for nuclear localization. The right panels show merged images of grazyme G and DAPI staining.

To verify the authenticity of the DDRT-PCR-derived cDNA sequences, gene-specific primers were designed based on published and experimentally derived sequences of the *granzyme G *gene for direct RT-PCR detection. Transcripts of the *granzyme G *gene were detected at the 2-cell stage, but were absent at the oocyte, 1-cell, 96 hr, and later stages (Figure 2A). The direct RT-PCR results were therefore consistent with the DDRT-PCR data. The spatial expression of *granzyme G *during developmental stages was subsequently examined and was determined to be confined to the extra-embryonic trophoblasts during the middle implantation stage (D10-14) of the mouse placenta (Figure 2B). The temporally constrained expression of *granzyme G *was further confirmed by whole-mount embryo *in situ *hybridization using a cloned *granzyme G *gene-specific fragment as a probe. The results consistently showed a high expression level at the 2-cell stage, which quickly diminished through the 4-cell stage (Figure 3). Furthermore, the granzyme G protein localized in the cytoplasm of 2-cell stage (Figure 4A) and 4-cell stage (Figure 4B) embryos, as demonstrated by whole-mount immunofluorescence. The protein completely disappeared by the 16-cell stage (Figure 4C).

### Antisense morpholino knock-down of granzyme G expression blocks embryos at the two-cell stage

**Figure 5:**
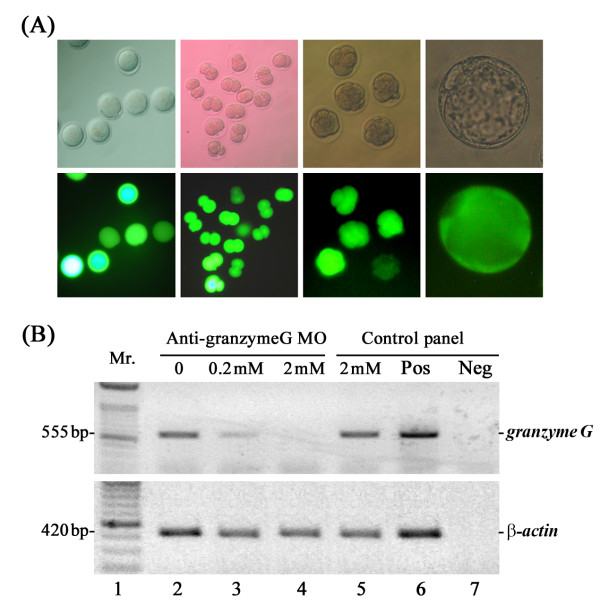
**Dosage and stability tests of antisense morpholinos in mouse embryos**. **(A) **The developmental potential of embryos was evaluated using 1-20 mM doses of control nonsense MO, which was conjugated to FITC fluorescent dye and microinjected into the cytoplasm of pronuclear-stage embryos. Upper panel: Phase contrast images. Lower panel: Fluorescence images. >Scale bar: 30 μm. **(B) ***Granzyme G *mRNA blocking efficiency test following microinjection of different doses of *granzyme G*-specific antisense MO (0, 0.2, and 2 mM) into 1-cell stage embryos. Total RNA was isolated from 2-cell stage experimental embryos, followed by RNase III digestion, purification, and subjection to RT-PCR analysis. In the control panel, mRNA extracted from the control MO-injected embryos was used as an un-blocked control (lane 5), mRNA from pregnant mouse uterine GMG cells was used as a positive control (Pos; lane 6), and reverse transcriptase-omitted mRNA template was used as a negative control (Neg; lane 7). The results are representative of three experiments.

To understand the function and importance of the *granzyme G *gene product during early embryonic stages, we introduced an antisense morpholino oligonucleotide (MO) against *granzyme G *into mouse embryos at the pronuclear stage. As a control, nonsense morpholino was conjugated to a FITC fluorophore and microinjected into 1-cell stage embryos. Preliminary tests showed that the control FITC-conjugated morpholino molecules distributed equally into dividing cells and were stably maintained until the blastocyst stage. Embryonic development was not affected after treatment with the control morpholino at doses between 1 and 20 mM (Figure 5A). When 2 mM *granzyme G *antisense morpholino was microinjected into the cytoplasm of 1-cell embryos, early development arrested at the 2- to 4-cell stages. Only 11.6% of treated embryos overcame 2-cell arrest and developed into 4-cell stage embryos. In contrast, the survival rate of embryos treated with 2 mM control morpholino, M2 buffer-injected embryos, and control *in vitro *cultured embryos was 93.3%, 85%, and 94.6%, respectively (Table 2).

**Table 2:** The effects of microinjection of different substrates into the cytoplasm of one-cell stage fertilized eggs on early embryo developmental potential

Different substrate treatment	Total embryo treated in one-cell stage^†^	No.(%) of survival embryo after treatment	No.(%) of embryos developed to				
			
			2-cell embryo	4-cell embryo	8-cell embryo	Morula embryo	Blastocyst embryo
2 mM granzyme G Morpholino	80	43(53.8)	39(90.7)^a^	5(11.6)^a^	0(0.0)^a^	0(0.0)^a^	0(0.0)^a^
2 mM control Morpholino	90	45(50.0)	45(100.0)^ab^	42(93.3)^b^	42(93.3)^b^	37(82.2)^b^	26(57.8)^b^
M2 buffer injection	46	20(43.5)	20(100.0)^ab^	17(85.0)^b^	17(85.0)^bc^	17(85.0)^b^	17(85.0)^c^
Control embryo (Uninjected)	74	--	74(100.0)^b^	70(94.6)^b^	57(77.0)^c^	57(77.0)^b^	57(77.0)^bc^

^† ^Six experiments were carried out in each group, and 10-15 embryos were used in each experiment.

^abc ^Different superscripts within a column indicate a statistically significant difference (p < 0.05).

### Developmental potential changes in response to granzyme G morpholino antisense treatment in a dose-dependent manner

Microinjection of serially diluted *granzyme G *morpholinos (2 mM, 0.2 mM, 0.02 mM, and 0.002 mM) into the cytoplasm of pronuclear mouse embryos resulted in survival rates to the blastocyst stage of 0%, 33.3%, 95.6%, and 87.2%, respectively (Table 3). The dose-response effect of the anti-*granzyme G *morpholino clearly correlated with the embryonic development potential.

**Table 3:** The developmental potential of mouse embryos cultured *in vitro *after microinjection of different concentrations of *granzyme G *antisense morpholinos into the cytoplasm of one-cell stage fertilized eggs

Dosage of Granzyme G Morpholino	**Total embryo treated in one-cell stage**^**†**^	No. (%) of survival embryo after treatment	No. (%) of embryos developed to				
			
			2-cell embryo	4-cell embryo	8-cell embryo	Morula embryo	Blastocyst embryo
2 mM	64	31(48.4)	25(80.7)^a^	7(22.6)^a^	0(0.0)^a^	0(0.0)^a^	0(0.0)^a^
0.2 mM	65	33(50.8)	32(97.0) ^b^	29(87.9)^b^	25(75.8)^b^	24(72.7)^b^	11(33.3)^b^
0.02 mM	81	45(55.6)	45(100.0)^b^	45(100.0)^c^	43(95.6)^c^	43(95.6)^c^	43(95.6)^c^
0.002 mM	63	39(61.9)	38(97.4)^b^	36(92.3)^bc^	34(87.2)^bc^	34(87.2)^bc^	34(87.2)^c^

^† ^Six experiments were carried out in each group, and 10-15 embryos were used in each experiment.

^abc ^Different superscripts within a column indicate a statistically significant difference (p < 0.05).

To determine the efficacy of the morpholino knock-down, the amount of *granzyme G *mRNA remaining after treatment was quantified. Total RNA isolated from different MO-treated 2-cell stage embryos was first digested with RNase III, followed by first-strand cDNA synthesis and semi-quantitative RT-PCR. For the pronuclear stage embryos injected with the intermediate dose (0.2 mM) of anti-*granzyme G *morpholino, the amount of intact *granzyme G *mRNA at the late 2-cell stage was reduced to 23% of the amount observed in normal untreated embryos, 2 mM control MO-treated embryos, and M2 buffer-injected embryos. For embryos treated with the high dose of *granzyme G *morpholino (2 mM), no *granzyme G *mRNA could be detected, presumably as a consequence of MO blocking and RNase III digestion (as shown in Figure 5B). We expect that this effect reflects the developmental arrest in 2-cell stage embryos.

**Figure 6:**
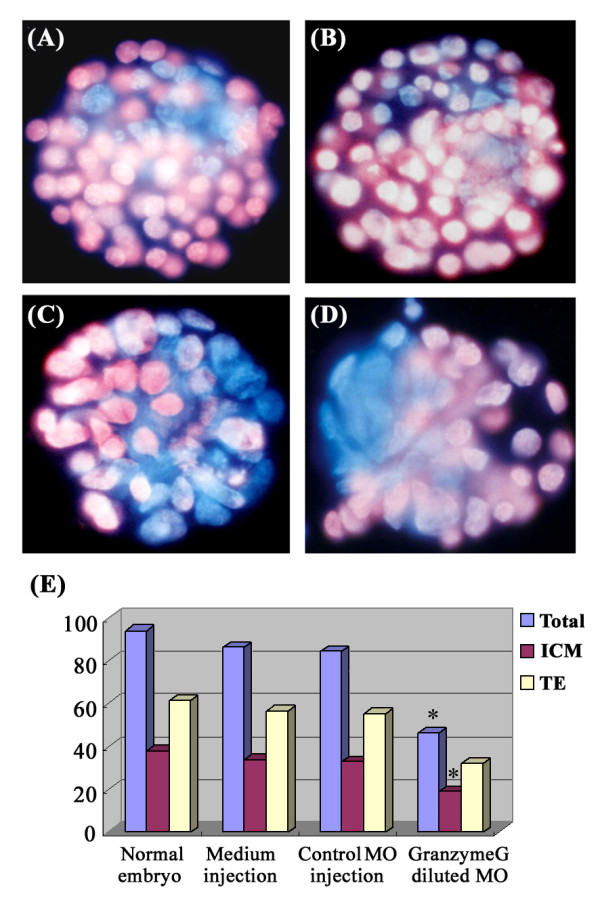
**The effect of inner cell mass (ICM) generation under low-dose anti-*granzyme G *morpholino treatment**. Differential blastomere staining of *in vitro *cultured blastocyst embryos at day 3.5: **(A) **normally developed embryo without MO treatment; **(B) **control nonsense MO-injected embryo; **(C, D) **low-dose anti-*granzyme G *specific morpholino- (0.2 mM) injected embryos. The trophectoderm (TE) cells are stained with pink fluorescence, and the ICM cells are stained with blue fluorescence. Scale bar: 30 μm. **(E) **Quantitative analysis of the numbers of total cells, ICM cells, and TE cells plotted according to the different treatments: normal blastocyst (n = 10), M2 medium-injected embryos (n = 8), control MO-injected embryos (n = 12), and low-dose anti-*granzyme G-*specific MO-injected embryos (n = 12).

Interestingly, the number of blastomere cells were significantly decreased in blastocysts treated with a low-dose of *granzyme G *morpholino (0.2 mM) when compared to normally developed blastocysts (43 ± 5 vs. 97 ± 8, p < 0.01; Figure 6). The inner cell mass (ICM) of treated blastocysts also exhibited a dispersed distribution compared to the localized ICM cluster of control blastocysts (Figure 6, A-D). Finally, the ICM of the 0.2 mM *granzyme G *MO-treated embryos also showed a dramatic decrease in differential staining compared to normally developed blastocysts (18 ± 5 vs. 37 ± 4, p < 0.05; Figure 6E).

### Maternal-zygotic transition inhibited by granzyme G knock-down

**Figure 7:**
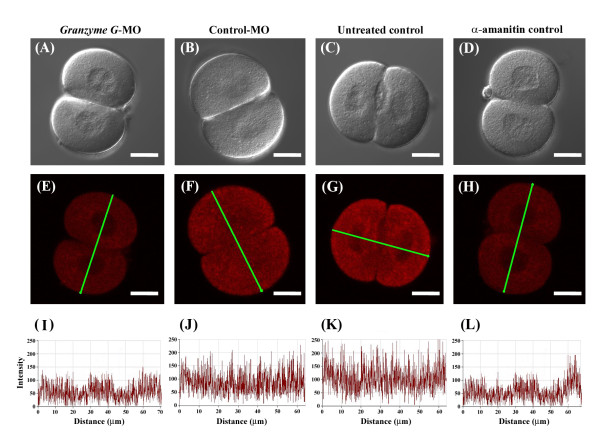
**Quantitation of zygotic RNA synthesis during the maternal-zygotic transition at the two-cell stage of the mouse embryo**. (A to D) Phase contrast images. (E to H) Images of a two-cell embryo treated with BrUTP and stained with Cy5-conjugated anti-BrdUTP antibody (red). (I to L) mRNA expression profiles of the green lines presented in panels E, F, G, and H, respectively. (A, E, I) Two-cell stage embryo following microinjection of *granzyme G *morpholino. (B, F, J) Two-cell stage embryo after injection of control morpholino. (C, G, K) Two-cell stage of a normal embryo. (D, H, L) Two-cell stage embryo pretreated with α-amanitin, a transcriptional inhibitor, as a negative control. Scale bar: 25 μm.

Bromouridine, which substitutes bromine to uridine, was used to trace zygotic gene expression during the maternal-zygotic transition (MZT). After its incorporation into cells, bromouridine is converted to Br-UTP. During transcription, converted Br-UTP is recognized as the same substrate as UTP and is incorporated into nascent RNAs. Confocal images of embryos were observed in bright field (Figure 7A-D) and under fluorescence (Figure 7E-H). Quantitative data showed that embryos microinjected with 2 mM *granzyme G *morpholino (Figure 7I) exhibited decreased zygotic gene transcription. Specifically, the newly synthesized zygotic RNA was less than 50% of the normal control embryos (Figure 7K). Moreover, the treatment of embryos with the control morpholino at the same concentration (Figure 7J) did not affect zygotic RNA synthesis compared to normal control embryos.

### The effect of serine protease inhibitors on embryo survival rate

**Figure 8:**
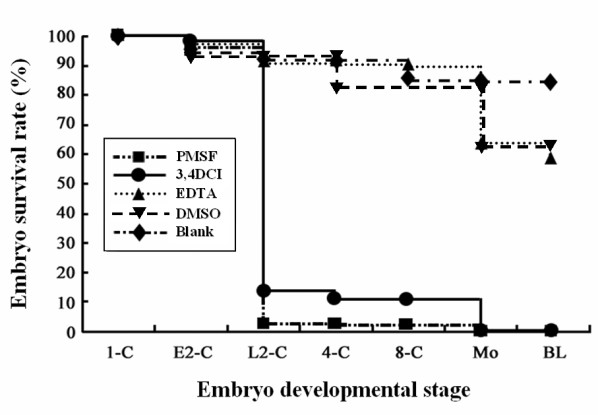
**Embryo survival rate to pre-implantation developmental stages under different protease inhibitor treatments**. Embryonic development potential was evaluated by adding 2 mM PMSF (n = 30) or 0.1 mM 3, 4-DCI (n = 28) for granzyme-specific serine protease inhibitors, 0.5 mM EDTA (n = 33) for non-granzyme-specific protease inhibitors, and 1% DMSO (n = 24) as a placebo control. A blank group (n = 30) that did not receive protease inhibitor was used to construct normal embryonic survival curves. The results are representative of three experiments.

Serine protease-specific inhibitors were used in the *in vitro *culture system to evaluate the effect of granzyme G activity suppression on embryonic development. The embryonic survival rate was dramatically reduced at the late 2-cell stage (3% and 12%) in the presence of 0.1 mM 3,4-dichloro-isocoumarin (3,4-DCI) and 2 mM phenyl methanesulphonyl fluoride (PMSF), respectively. Due to the short life of PMSF activity, three different stages (pronuclear, 2-cell, and 8-cell) of mouse embryos were supplemented one time with 2 mM PMSF in the culture medium. PMSF inhibits survival only at the 2-cell stage, when granzyme G exhibits serine protease activity in the embryos. However, the addition of other kinds of metalloproteinase inhibitors, such as 0.5 mM EDTA, had no obvious consequence, as survival rates of > 90% to the late 2-cell stage and 60% to the blastocyst stage were observed (Figure 8). The addition of 1% DMSO solvent, as a vehicle control, did not interfere wuth mouse embryo survival, especially in the maternal-to-zygotic transition at the 2-cell stage (Figure 8).

## Discussion

Normal embryonic development, as well as many genetic disorders and cancers, involves a delicate balance between the processes of cellular differentiation and proliferation. Many developmental genes have been identified as candidate genes for cancers or other forms of human disorders, and may be developed into novel diagnostic markers or new therapeutic targets. Early-expressed genes like those encoding the homeobox and zinc-finger proteins play central roles in the regulation of gene cascades and signal transduction pathways, and thus the downstream effects of these pathways [21,22]. Thus, the primary goal of this study was to identify genes that are differentially expressed at the pre-implantation stages of embryonic development to establish novel candidates for use in these broader applications. We then assembled a comprehensive spatial and temporal expression profile of the most promising genes using DDRT-PCR (Figure 1). DDRT-PCR allows the systematic comparison of the expression of all mRNAs among several cell populations. It has been used to address biological questions in mammalian systems, including cell differentiation, cell activation, cell stress, and identification of drug targets [23]. The advantages of DDRT-PCR over the traditional subtractive hybridization and other comparative RNA techniques include (i) rapidity and simplicity of assays, (ii) small quantities of RNA, (iii) increased sensitivity, (iv) reproducibility, (v) ability to identify differentially expressed genes in more than one population, and (vi) ability to compare several cell populations or variables simultaneously. However, the assay does have its limitations, including false-positive results, the inability to confirm differential expression, and a reduced output compared to cDNA microarrays.

It is an interesting possibility that differential display genes in pre-implantation embryos may help to "pull the trigger" and initiate embryonic development and differentiation. At the pre-implantation stages of embryogenesis, mammalian embryos undergo a so-called "zygotic gene activation" or "embryonic gene activation". During this period, the activity of the embryonic genome changes significantly, and the nuclei begin to produce many new species of mRNA. Several important genes listed in Table 1 are targets for functional studies in early embryonic development. For example, the *Snf2 h *gene, which is expressed in 4-cell stage embryos, encodes a protein with ISWI ATPase activity. A previous report has demonstrated that null *Snf2h*^-/- ^embryos died during the preimplantation stage [24]. Blastocyst outgrowth experiments indicated that the loss of Snf2 h results in growth arrest and cell death of both the trophectoderm and inner cell mass. In the present study, we successfully identified tweenty-three differentially expressed genes in early mouse embryos (Table 1). Among them, the *granzyme G *transcript encoding a serine protease was expressed at the 2-cell stage (Figures 2 and 3). It was previously reported that the differentiation of mouse GMG cells, which begins at day 7 of gestation, involves the accumulation of cytolytic mediators, including perforin and the serine protease granzymes A-H, within cytoplasmic granules [19]. GMG cells have been proposed to regulate trophoblast invasion into maternal deciduas. Indeed, trophoblast killing by murine and human uterine NK cells has been reported [20]. Other previously proposed functions of granzyme G include (i) the lysis of virus-infected cells present in the uterus and placenta, (ii) the initiation of abortion, (iii) the destruction of the extracellular matrix and cells at the placenta/uterine interface to promote parturition, (iv) nutritive functions, and (v) cytokine production [14,25]. However, there have been no reports of the expression of granzyme G in 2-cell mouse embryos or any conclusive data supporting a particular biological function.

To determine the function of specific genes in the field of developmental biology, many effective procedures have been advanced to regulate or disrupt gene expression. In early mouse embryos, researchers have used a variety of methods, including antisense RNA, double-stranded RNA interference, and gene knock-out approaches [26,27] to block target gene expression and assess the phenotype resulting from the deficiency. Antisense RNA is one of most common methods used to down-regulate mRNA translation, especially in culture systems. However, the potential of this technique was unfulfilled until recently because the field was dominated by the use of phosphorothioates, which are plagued by unpredictable targeting, sensitivity to nucleases, poor sequence specificity, and a host of non-antisense effects [28]. The use of morpholino oligonucleotides (MO) to suppress the translation of targeted genes is proving to be a valuable tool for investigations of gene function in zebrafish [29], *Xenopus *[30], and sea urchins [31]. In mice, MOs have been applied to pre-implantation mouse embryos using the ethoxylated polyethylenimine (EPEI) method [32] and in the germinal vesicle stage of mouse oocytes by microinjection [33]. Both methods successfully suppressed target gene expression. We used this well-defined procedure to investigate the function of granzyme G during pre-implantation development in mouse embryos. In our preliminary test, control FITC-conjugated morpholino molecules that were microinjected into 1-cell stage embryos were equally distributed into dividing cells and stably retained until the blastocyst stage (Figure 5).

In the non-specific MO toxicity test, the results showed that embryonic development was not affected by the introduction of a wide range of doses (1-20 mM) of control morpholinos. Using the MO technique, we further found that 2 mM *granzyme G*-specific MO inhibited blastocyst formation in 100% of the treated embryos (Table 2). The knock-down efficiency of the MO treatment was measured using the RNase III protection assay [34]. RNase III is a RNA endonuclease that specifically cleaves double-stranded RNAs, including the complex formed by the antisense morpholino and the endogenous sense mRNA, into small fragments. Semi-quantitative RT-PCR analysis revealed that the degradation rates of *granzyme G *mRNA were 100%, 77%, and 0% after microinjection of pronuclear stage embryos with 2 mM, 0.2 mM, and 0 mM of anti-*granzyme G *morpholino, respectively (Figure 5B). In addition, the microinjection of a serial dilution of *granzyme G *morpholino into the cytoplasm of pronuclear mouse embryos showed a dose-dependent inhibition of blastocyst development rates (Table 3).

Other kinds of serine proteases have been shown to be expressed in pre-implantation embryos, such as the seven-member family of subtilisin-like, calcium-dependent serine proteases known as proprotein convertases (PCs) [35]. In early embryos, PCs cleave a wide variety of precursors into secreted peptides and proteins, including hormones, neuropeptides, growth factors, enzymes, membrane receptors, and extracellular matrix proteins [36]. They have been implicated in most biological processes affecting growth, development, and physiology [37]. Indeed, several PC substrates are expressed in pre-implantation embryos, such as precursors to gonadotropin-releasing hormone (GnRH), transforming-growth factors (TGF) β-1 and β-2, platelet-derived growth factor (PDGF), and insulin-like growth factor 1 receptor (IGF1-R) [38,39]. Among these, GnRH has been shown to be critical for normal pre-implantation embryonic growth. *ProGnRH *mRNA and the GnRH peptide are detectable in pre-implantation embryos from the morula to the blastocyst stage [40]. Antisense inhibition of *GnRH *mRNA translation has been shown to block the development of these embryos *in vitro *[38]. As proprotein convertase 1 (PC1) is the most likely proGnRH convertase, it too may be important for pre-implantation development at the morula stage, whereas granzyme G appears to act in the maternal-zygotic transition at the 2-cell stage. As shown in the BrUTP incorporation experiment (Figure 7), treatment of embryos with *granzyme G*-specific MO, as well as the transcription inhibitor α-amanitin, inhibited RNA synthesis during the maternal-to-zygotic transition.

To determine the effects of blocking granzyme G activity in pre-implantation mouse embryos, we exposed the developing embryos to three known protease inhibitors: two serine protease-specific inhibitors and one non-serine protease inhibitor [41,42]. Only the serine protease-specific inhibitors, 3,4-DCI and PMSF, phenocopied the MO-induced effect, blocking the maternal-zygotic transition and dramatically decreasing the embryo survival rate at the late 2-cell stage. However, the metalloproteinase inhibitor, EDTA, had no such effect (Figure 8). The effect of PMSF on different stages of development was further evaluated in this study. Due to the short life of PMSF activity, three different stages of mouse embryos were separated and supplemented one time with 2 mM PMSF in the culture medium. The inhibition time window is only at the 2-cell stage, which is the stage at which granzyme G functions as an active serine protease in the embryos.

**Figure 9:**
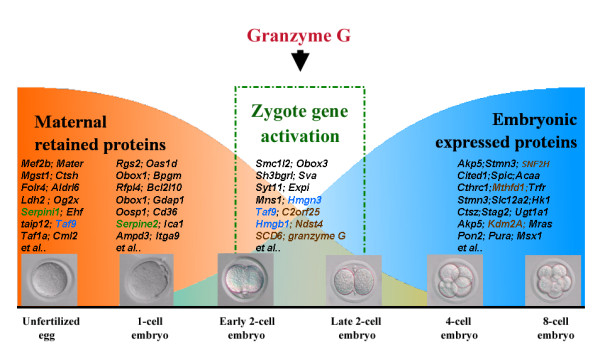
**Proposed model for granzyme G action during mouse early embryonic development**. Maternally produced proteins are abundant in fertilized mouse eggs up to the early 2-cell stage, after which zygotic gene expression increases. Granzyme G, encoding a serine protease, may contribute to this maternal-zygote exchange process. The genes marked with blue color were simultaneously presented in our gene list (Table1) and in the previous report identified by cDNA microarray [49]. See the text for details.

Oocytes, the female germ cells, contain all of the messenger RNAs necessary to start a new life, but typically wait until fertilization to begin development. The transition from oocyte to zygote involves many changes, including protein synthesis, protein and RNA degradation, organelle remodeling, and even the onset of sexual differentiation [43-45]. Fifteen to thirty percent of mRNA transcripts are degraded during zygote gene activation [46]. Proteins are also targeted for degradation during the oocyte-to-zygote transition [47]. Protein degradation often serves to inactivate proteins that are needed early in the transition but that would be harmful later [46]. Previous reports have demonstrated that two serine protease inhibitors, Serpini 1 and Serpine 2, are temporally expressed in the unfertilized egg and 1-cell stage embryo, respectively [48,49]. These inhibitors may prevent granzyme G function until the 2-cell stage, where it regulates the maternal-zygotic transition. A possible role for granzyme G in the maternal-zygotic transition process is to participate in maternal protein degradation and the subsequent initiation of zygotic gene expression, as proposed in Figure 9. Dependence on granzyme G activity was first observed at the early 2-cell stage and persisted until the late 2-cell stage. This responsiveness of the mouse zygote appears to require new transcription from the zygotic genome [45].

## Conclusion

This study has clearly demonstrated the feasibility of the application of DDRT-PCR for the identification of transcripts that are differentially expressed in mouse embryos at various pre-implantation developmental stages. Numerous novel transcripts have been identified at the unfertilized egg, 2-cell, and 4-cell embryonic stages. We characterized for the first time the expression of *granzyme G *during early stage embryogenesis. Overall, our results suggest that granzyme G is an important factor in early mouse development and may play a novel role in the elimination of maternal proteins and the triggering of zygotic gene expression during the maternal-zygotic transition. Future studies will attempt to determine the specific function and regulation of these genes to enhance our understanding of normal developmental events in very early embryos.

## Methods

### Collection of unfertilized mouse eggs and embryos at various stages of early development

Eggs were collected from female ICR mice that were intraperitoneally injected with 10 IU of pregnant mare serum gonadotrophin (PMSG, China Chemical & Pharmaceutical Co., Taiwan) to induce superovulation. After 48 hr, the mice were injected with 10 IU of human chorionic gonadotrophin (hCG, China Chemical & Pharmaceutical Co., Taiwan). All unfertilized oocytes were collected 14-15 hr after the hCG injection in phosphate buffered saline (PBS) containing 200 IU/ml of hyaluronidase, to disperse cumulus cells, and were then transferred into human tubule fluid (HTF) medium (Irvine Scientific, Santa Ana, CA). For the collection of fertilized eggs, the superovulation-induced females were mated with male ICR mice. The presence of a vaginal plug on the subsequent morning (day 1) signified successful mating, and time post-hCG was used to measure the developmental age of the embryos. Embryos were flushed from the oviducts using PBS buffer at 48 hr post-hCG for the collection of 2-cell embryos and at 60 hr post-hCG for the collection of 4-cell embryos. The collected embryos were washed three times with PBS containing hyaluronidase and were then transferred into HTF medium for further *in vitro *embryo culture or for the extraction of total RNA [50]. The animal use protocol in this study has been reviewed and approved by the Institutional Animal Care and Use Committee of the National Chung Hsing University (IACUC Approval number: 96-52).

### RNA preparation and DDRT-PCR assay

Total RNA was prepared from pools of about 100 embryonic cells using the previously described guanidinium acid-phenol method [51]. Isolated total RNA was first subjected to reverse transcription (RT) using one of the three anchor primers (H-T11-C, -G, or -A) and the Superscript II RT kit (Gibco BRL, Grand Island, NY). The quality of the total RNA and cDNAs were examined using amplification of the house-keeping gene, β-*actin*, as described [52]. This was followed by 35 thermal cycles of PCR using the original anchor primer and one of the eight random primers, as shown in the manufacturer's description of the differential display RNAimage system (GenHunter Co., Nashville, TN), in the presence of 2 μCi [α-^33^P] dCTP (Amersham Bioscience, Amersham, UK). PCR conditions were as follows: 94°C for 30 sec, 32°C for 1.5 min, and 70°C for 1.5 min. The DDRT-PCR products were electrophoresed in 8% denaturing polyacrylamide gels of 0.2-mm thickness. After drying on a 3 MM filter, the gels were autoradiographed [52,53].

### Sequence analysis, RT-PCR confirmation, and ribonuclease protection assay

For re-amplification, bands of interest were excised, and DNA was eluted from the gel by incubation of the gel slice at 70°C in deionized water. PCR was performed using aliquots of the recovered DNA fragment and the same combination of anchor and random primers. The PCR products were cloned into pGEM-T vector (Invitrogen, Carlsbad, CA) and sequencing was performed using an automatic sequencer 373A (Applied Biosystems Inc., Foster, CA). The differential display mRNA bands were further confirmed by individual RT-PCR assays. The specific primers employed for *granzyme G *gene expression analysis were synthesized as follows: 5'-GAT TCT CCT GAC CCT ACT TC-3' (forward) and 5'-CTG CGT GGT CTT GGA ATA GG-3' (reverse). The β-actin transcript was used as an internal RT-PCR control and for the semi-quantitative quantitation of the *granzyme G *RNA level as described previously [54,55]. The ribonuclease protection assay (RPA) was performed by first generating antisense RNA probes using the MAXIscript *in vitro *transcription kit (Ambion, Austin, TX) and the cloned DDRT-PCR products in the presence of [α-^32^P]UTP. The labeled gene-specific antisense RNA was mixed with total RNA, and RPA analysis was carried out using a commercial RPAII kit (Ambion, Austin, TX).

### Morpholino oligonucleotide synthesis and microinjection

The anti-*granzyme G *morpholino oligonucleotide (MO) was designed and synthesized (Gene Tools, LLC, Philomath, OR) to be complementary to 25 bases of the mouse *granzyme G *mRNA, lying downstream of the initiation codon and containing the Kozak sequence. The sequence of this MO was 5'-ATC AGG ATT GGT GGC ATC TTC CCA G-3'. A fluorescent FITC-labeled control nonsense MO with a random sequence, 5'-CCT CTT ACC TCA GTT ACA ATT TAT A-3', was used to measure the MO distribution after cytoplasmic injection using Pieo (PMM 150FU, Prime Tech, Japan) and to control for non-specific MO toxicity. In each experimental trial, an additional control group was included that consisted of embryos treated only with M2 medium. Experimental groups often included embryo pools that were not injected with MO or M2 medium and served as normal controls. All experiments were conducted a minimum of three times using embryos collected from a minimum of three separate embryo flushes.

### Whole-mount in situ hybridization

The process of whole mount in situ hybridization of mouse embryos was modified from Lefebvre et al. [56]. The zona pellucida was removed with Tyrode acid solution (pH 2.5) (Sigma, St. Louis, MO), and the embryos were fixed in 4% formaldehyde, 10% acetic acid, 1 × PBS (100 mM Na2HPO4, 20 mM KH2PO4, 137 mM NaCl, 27 mM KCl, pH 7.4) on a 0.1% polylysine-coated cover slide for 30 min. After two PBS washes, the embryos were permeabilized by treatment with 70% ethanol overnight at 4°C. Following fixation, embryos were rehydrated for 5 min at room temperature in 2 × SSC (300 mM NaCl, 30 mM sodium citrate, pH 7.0), 50% formamide and then were hybridized for 2-3 hr at 37°C in 200 μl of a mixture containing 10% dextran sulfate, 2 mM vanadyl-ribonucleoside complex, 0.02% RNAse-free BSA, 40 μg *E. coli *tRNA, 2 × SSC, 15% formamide, and 50 ng of *granzyme G *antisense oligonucleotide probes. After hybridization, embryos were washed twice for 30 min at the appropriate stringency (2 × SSC, 50% formamide at 37°C). For DNA staining, embryos were stained with 1 μg/mL Hoechst 33342 (Bisbenzimid, Sigma, St. Louis, MO) in PBS for 5 min and washed twice with 1 × PBS. Cover slides were dried and then transferred to the slide on a drop of mounting oil. The excess mounting oil was aspirated, and the cover slide was sealed with nail polish. The slides were stored at 4°C or immediately observed using a fluorescence microscope [55] or a confocal microscope (LSM50, Carl Zeiss Meditec, Dublin, CA) [57].

### Whole-mount immunofluorescence staining

The protocol of immunofluorescence staining was modified from our previous report [53]. Briefly, different stages of mouse embryos were mounted on a 0.1% polylysine-coated cover slide and stained with goat anti-granzyme G antibody (sc-103533, Santa Cruz Biotech. Inc., Santa Cruz, CA), which was diluted 1:1000 with antibody dilutant buffer (1% BSA and 0.1% Triton-X 100 in PBS), followed by a secondary antibody of rabbit anti-goat IgG-FITC. After washing, the stained embryos were observed with a Carl Zeiss LSM5l0 confocal laser scanning microscope with a 63 × objective lens.

### Differential staining of inner cell mass (ICM) and trophectoderm cells (TE)

To determine the total cell numbers and the ratio of ICM to TE cells in *granzyme G *antisense MO-treated (sub-inhibition dose) embryos, a differential staining procedure was performed using a representative sample of day 3.5 blastocysts, as previously described [58] with minor modifications. Briefly, after removal of the zona pellucida with Tyrode acid solution (pH 2.5), the embryos were incubated in trinitrobenzene-sulfonic-acid (TNBS, Sigma, St. Louis, MO) for 10 min on ice, washed in M2 medium, and incubated in 0.1 mg/ml anti-dinitrophenyl-BSA (anti-DNP-BSA; ICN Biochemicals, Cleveland, OH) at 37°C for 10 min. After washing in PBS+PVA, complement lysis was induced by incubating the embryos in guinea pig complement (Sigma, St. Louis, MO), diluted 1:4 in PBS+PVA and supplemented with 10 μg/ml propidium iodide (Sigma, St. Louis, MO), at 37°C for 20 min, followed by brief washing and fixation in ice-cold ethanol. The inner nuclei were stained with 0.05 mM Hoechst 33342 for 10 min. Stained embryos were then mounted in 100% glycerol. Under a fluorescence microscope, the outer TE cells were identified by the pink fluorescence of propidium iodide, while the ICM cells were recognized by the blue fluorescence of the bisbenzimide.

### In vivo RNA synthesis assay

A published protocol was used with some modifications [59]. After *granzyme G*-MO, control-MO, or α-amanitin treatment of 2-cell stage fertilized eggs (n = 20), bromouridine (BrU; Sigma, St. Louis, MO) was added at 100 μM for 12 hr to label newly synthesized zygotic RNA. The embryos were washed twice with 1 × PBS and subjected to whole-mount in situ staining using Cy5-conjugated anti-BrdU antibody (Roche Diagnostic). The fluorescence quantification was performed using the software for the confocal microscope (LSM50, Carl Zeiss Meditec, Dublin, CA)

### Statistical Analyses

Experiments were repeated at least three times with embryo collection replicates. The collected data were subjected to a chi-squared analysis using the statistical analysis system, SAS, for multiple comparisons. Differences of p < 0.05(*) and p < 0.01 (**) were considered statistically significant.

## Abbreviations

3,4-DCI: 3,4-dichloroisocoumarin; DDRT-PCR: differential display reverse transcription-polymerase chain reaction; EPEI: ethoxylated polyethylenimine; GnRH: gonadotropin-releasing hormone; hCG: human chorionic gonadotrophin; HTF: human tubule fluid; MO: morpholino oligonucleotide; MTHF: methylenetetrahydrofolate; MZT: maternal-zygotic transition; PMSF: phenyl methanesulphonyl fluoride; PMSG: pregnant mare serum gonadotrophin; TBP: TATA-box binding protein; TNBS: trinitrobenzene-sulfonic-acid.

## Authors' contributions

TCT, WL, EHC, and SHY handled the embryo culture and developed the study protocol; all authors participated in the study conduct; WTKC, MSL, and KYC contributed to the critical granzyme G knock-down assay; TCT, SHY, and CMC collected the data and drafted the paper. All authors read and approved the final manuscript.

## References

[B1] Schultz RM The molecular foundations of the maternal to zygotic transition in the pre-implantation embryo Hum Reprod Update 2002 8 323 31 10.1093/humupd/8.4.323 12206467

[B2] Wu X Viveiros MM Eppig JJ Bai Y Fitzpatrick SL Matzuk MM Zygote arrest 1 (Zar1) is a novel maternal-effect gene critical for the oocyte-to-embryo transition Nat Genet 2003 33 187 91 10.1038/ng1079 12539046

[B3] Lee KF Chow JFC Xu JS Chan STH Ip SM Yeung WSB A comparative study of gene expression in murine embryos developed in vivo, cultured in vitro, and co-cultured with human oviductal cells using messenger ribonucleic acid differential display Biol Reprod 2001 64 910 7 10.1095/biolreprod64.3.910 11207208

[B4] Thompson EM Legouy E Christians E Renard JP Progressive maturation of chromatin structure regulates HSP70.1 gene expression in the pre-implantation mouse embryo Development 1995 121 3425 37 7588075 10.1242/dev.121.10.3425

[B5] Latham KE Rambhatla L Hayashizaki Y Chapman VM Stage-specific induction and regulation by genetic imprinting of the imprinted mouse *U2afbp-rs *gene in the pre-implantation mouse embryo Dev Biol 1995 168 670 6 10.1006/dbio.1995.1111 7729597

[B6] Davis WJ De Sousa PA Schultz RM Transient expression of translation initiation factor eIF-4C during the 2-cell stage of the pre-implantation mouse embryo: identification by mRNA differential display and the role of DNA replication in zygotic gene activation Dev Biol 1996 174 190 201 10.1006/dbio.1996.0065 8631492

[B7] Worrad DM Turner BM Schultz RM Temporally restricted spatial localization of acetylated isoforms of histone H4 and RNA polymerase II in the 2-cell mouse embryo Development 1995 121 2949 59 7555721 10.1242/dev.121.9.2949

[B8] Liang P Pardee AB Differential display of eukaryotic messenger RNA by means of the polymerase chain reaction Science 1992 257 967 71 10.1126/science.1354393 1354393

[B9] Gupta R Thomas P Beddington RSP Rigby PWJ Isolation of developmentally regulated genes by differential display screening of cDNA libraries Nucl Acids Res 1998 26 4538 9 10.1093/nar/26.19.4538 9742260 PMC147855

[B10] Zimmermann JW Schultz RM Analysis of gene expression in the pre-implantation mouse embryo: use of mRNA differential display Proc Natl Acad Sci USA 1994 91 5456 60 10.1073/pnas.91.12.5456 7515503 PMC44014

[B11] Ibrahim MM Razmara M Nguyen D Donahue RJ Wubah JA Knudsen TB Altered expression of mitochondrial 16 S ribosomal RNA in p53-deficient mouse embryos revealed by differential display Biochim Biophys Acta 1998 1403 254 64 10.1016/S0167-4889(98)00066-4 9685670

[B12] Jenne DE Tschopp J Granzymes, a family of serine proteases released from granules of cytolytic T lymphocytes upon T cell receptor stimulation Immunol Rev 1988 103 53 77 10.1111/j.1600-065X.1988.tb00749.x 3292396

[B13] Allen MP Nilsen-Hamilton M Granzymes D, E, F, and G are regulated through pregnancy and by IL-2 and IL-15 in granulated metrial gland cells J Immunol 1998 161 2772 9 9743335

[B14] Tarachand U Decidualisation: origin and role of associated cells Biol Cell 1986 57 9 16 2945613 10.1111/j.1768-322x.1986.tb00459.x

[B15] Guimond M Luross JA Wang B Terhorst C Danial S Croy BA Absence of natural killer cells during murine pregnancy is associated with reproductive compromise in TgE26 mice Biol Reprod 1997 56 169 79 10.1095/biolreprod56.1.169 9002646

[B16] Guimond M Wang B Fujita J Terhorst C Croy BA Pregnancy-associated uterine granulated metrial gland cells in mutant and transgenic mice Am J Reprod Immunol 1996 35 501 9 8792932 10.1111/j.1600-0897.1996.tb00049.x

[B17] Linnemeyer PA Pollack SB Stage-specific expression of activation antigens on NK cells at uterine implantation sites of mice J Immunol 1994 153 1478 86 8046227

[B18] Starkey PM Expression on cells of early human pregnancy decidua, of the p75, IL-2 and p145, IL-4 receptor proteins Immunology 1991 73 64 70 2045128 PMC1384519

[B19] King A Loke YW Human trophoblast and JEG choriocarcinoma cells are sensitive to lysis by IL-2-stimulated decidual NK cells Cell Immunol 1990 129 435 48 10.1016/0008-8749(90)90219-H 1696527

[B20] Stewart I Mukhtar DD The killing of mouse trophoblast cells by granulated metrial gland cells in vitro Placenta 1988 9 417 25 10.1016/0143-4004(88)90054-9 3211872

[B21] Lim H Ma L Ma WG Maas RL Dey SK Hoxa-10 regulates uterine stromal cell responsiveness to progesterone during implantation and decidualization in the mouse Mol Endocrinol 1999 13 1005 17 10.1210/me.13.6.1005 10379898

[B22] Khalfallah O Faucon-Biguet N Nardelli J Meloni R Mallet J Expression of the transcription factor Zfp191 during embryonic development in the mouse Gene Expr Patterns 2008 8 148 54 10.1016/j.gep.2007.11.002 18096443

[B23] Kim SH Hong KO Chung WY Hwang JK Park KK Abrogation of cisplatin-induced hepatotoxicity in mice by xanthorrhizol is related to its effect on the regulation of gene transcription Toxicol Appl Pharmacol 2004 196 346 55 10.1016/j.taap.2003.11.020 15094305

[B24] Stopka T Skoultchi AI The ISWI ATPase Snf2 h is required for early mouse development Proc Natl Acad Sci USA 2003 100 14097 102 10.1073/pnas.2336105100 14617767 PMC283552

[B25] Croy BA Granulated metrial gland cells: hypotheses concerning possible functions during murine gestation J Reprod Immunol 1994 27 85 94 10.1016/0165-0378(94)90025-6 7884744

[B26] Stein P Svoboda P Schultz RM Transgenic RNAi in mouse oocytes: a simple and fast approach to study gene function Dev Biol 2003 256 187 93 10.1016/S0012-1606(02)00122-7 12654301

[B27] Stebbins-Boaz B Richter JD Translational control during early development Crit Rev Eukary Gene Expr 1997 7 73 94 10.1615/critreveukargeneexpr.v7.i1-2.50 9034716

[B28] Schepers U RNA interference in practice 2005 John Wiley and Sons Ltd. Chichester, UK 261 99

[B29] Ahn DG Kourakis MJ Rohde LA Silver LM Ho RK T-box gene *tbx5 *is essential for formation of the pectoral limb bud Nature 2002 417 754 8 10.1038/nature00814 12066188

[B30] Akagi K Park EK Mood K Daar IO Docking protein SNT1 is a critical mediator of fibroblast growth factor signaling during *Xenopus *embryonic development Dev Dyn 2002 223 216 28 10.1002/dvdy.10048 11836786

[B31] Angerer LM Oleksyn DW Levine AM Li X Klein WH Angerer RC Sea urchin goosecoid function links fate specification along the animal-vegetal and oral-aboral embryonic axes Development 2001 128 4393 404 11714666 10.1242/dev.128.22.4393

[B32] Siddall LS Barcroft LC Watson AJ Targeting gene expression in the pre-implantation mouse embryo using morpholino antisense oligonucleotides Mol Reprod Dev 2002 63 413 21 10.1002/mrd.10202 12412042

[B33] Coonrod SA Bolling LC Wright PW Visconti PE Herr JCA A morpholino phenocopy of the mouse mos mutation Genesis 2001 30 198 200 10.1002/gene.1065 11477708

[B34] Lamontagne B Larose S Boulanger J Elela SA The RNase III family: a conserved structure and expanding functions in eukaryotic dsRNA metabolism Curr Issues Mol Biol 2001 3 71 8 11719970

[B35] Seidah NG Chretien M Proprotein and prohormone convertases: a family of subtilases generating diverse bioactive polypeptides Brain Res 1999 848 45 62 10.1016/S0006-8993(99)01909-5 10701998

[B36] Zhou A Webb G Zhu X Steiner DF Proteolytic processing in the secretory pathway J Biol Chem 1999 274 20745 8 10.1074/jbc.274.30.20745 10409610

[B37] Taylor NA Van de Ven WJ Creemers JW Curbing activation: proprotein convertases in homeostasis and pathology FASEB J 2003 17 1215 27 10.1096/fj.02-0831rev 12832286

[B38] Raga F Casan EM Kruessel J Wen Y Bonilla-Musoles F Polan ML The role of gonadotropin-releasing hormone in murine pre-implantation embryonic development Endocrinology 1999 140 3705 12 10.1210/en.140.8.3705 10433230

[B39] Chow JF Lee KF Chan ST Yeung WS Quantification of transforming growth factor beta1 (TGFbeta1) mRNA expression in mouse pre-implantation embryos and determination of TGFbeta receptor (type I and type II) expression in mouse embryos and reproductive tract Mol Hum Reprod 2001 7 1047 56 10.1093/molehr/7.11.1047 11675471

[B40] Casan EM Raga F Polan ML GnRH mRNA and protein expression in human pre-implantation embryos Mol Hum Reprod 1999 5 234 9 10.1093/molehr/5.3.234 10333357

[B41] Woodard SL Fraser SA Winkler U Jackson DS Kam CM Powers JC Hudig D Purification and characterization of lymphocyte chymase I, a granzyme implicated in perforin-mediated lysis J Immunol 1998 160 4988 93 9590247

[B42] Drapkin PT Monard D Silverman AJ The role of serine proteases and serine protease inhibitors in the migration of gonadotropin-releasing hormone neurons BMC Dev Biol 2002 2 1 10.1186/1471-213X-2-1 11872147 PMC65692

[B43] Kobayashi S Fujihara Y Mise N Kaseda K Abe K Ishino F Okabe M The X-linked imprinted gene family Fthl17 shows predominantly female expression following the two-cell stage in mouse embryos Nucleic Acids Res 2010 in press 20185572 10.1093/nar/gkq113 PMC2887969

[B44] Léandri RD Archilla C Bui LC Peynot N Liu Z Cabau C Chastellier A Renard JP Duranthon V Revealing the dynamics of gene expression during embryonic genome activation and first differentiation in the rabbit embryo with a dedicated array screening Physiol Genomics 2009 36 98 113 19001509 10.1152/physiolgenomics.90310.2008

[B45] Stizel ML Seydoux G Regulation of the oocyte-to-zygote transition Science 2007 316 407 8 10.1126/science.1138236 17446393

[B46] Wang QT Piotrowska K Ciemerych MA Milenkovic L Davis RW Zernicka GM A genome-wide study of gene activity reveals developmental signaling pathways in the preimplantation mouse embryo Dev Cell 2004 6 133 44 10.1016/S1534-5807(03)00404-0 14723853

[B47] Evsikov AV Graber JH Brockman JM Hampl A Holbrook AE Singh P Eppig JJ Solter D Knowles BB Cracking the egg: molecular dynamics and evolutionary aspects of the transition from the fully grown oocyte to embryo Genes Dev 2006 20 2713 27 10.1101/gad.1471006 17015433 PMC1578697

[B48] Hamatani T Carter MG Sharov AA Ko MS Dynamics of global gene expression changes during mouse preimplantation development Dev Cell 2004 6 117 31 10.1016/S1534-5807(03)00373-3 14723852

[B49] Zeng F Baldwin DA Schultz RM Transcript profiling during preimplantation mouse development Dev Biol 2004 272 483 96 10.1016/j.ydbio.2004.05.018 15282163

[B50] Chen CM Wang CH Wu SC Lin CC Lin SH Cheng WTK Temporal and spatial expression of biologically active human factor VIII in the milk of transgenic mice driven by mammary-specific bovine α-lactalbumin regulation sequences Transgenic Res 2002 11 257 68 10.1023/A:1015651302674 12113458

[B51] Chomczynski P Sacchi N Single-step method of RNA isolation by acid guanidinium thiocyanate-phenol-chloroform extraction Anal Biochem 1987 162 156 9 10.1016/0003-2697(87)90021-2 2440339

[B52] Chen HL Wang LC Chang CH Yen CC Cheng WT Wu SC Hung CM Kuo MF Chen CM Recombinant porcine lactoferrin expressed in the milk of transgenic mice protects neonatal mice from a lethal challenge with enterovirus type 71 Vaccine 2008 26 891 8 10.1016/j.vaccine.2007.12.013 18207613

[B53] Wu SC Chen HL Yen CC Kuo MF Yang TS Wang SR Weng CN Chen CM Cheng WT Recombinant porcine lactoferrin expressed in the milk of transgenic mice enhances offspring growth performance J Agri Food Chem 2007 55 4670 7 10.1021/jf063759o 17489602

[B54] Chen CM Chen HL Hsiau TH Hsiau AH Shi H Brock GJ Wei SH Caldwell CW Yan PS Huang TH Methylation target array for rapid analysis of CpG island hypermethylation in multiple tissue genomes Am J Pathol 2003 163 37 45 12819009 10.1016/S0002-9440(10)63628-0 PMC1868173

[B55] Chen HL Yen CC Lu CY Yu CH Chen CM Synthetic porcine lactoferricin with a 20-residue peptide exhibits antimicrobial activity against *Escherichia coli*, *Staphylococcus aureus*, and *Candida albicans* J Agric Food Chem 2006 54 3277 82 10.1021/jf053031s 16637685

[B56] Lefebvre C Terret ME Djiane A Rassinier P Maro B Verlhac MH Meiotic spindle stability depends on MAPK-interacting and spindle-stabilizing protein (MISS), a new MAPK substrate J Cell Biol 2002 157 603 13 10.1083/jcb.200202052 12011110 PMC2173866

[B57] Yen CC Lin CY Chong KY Tsai TC Shen CJ Lin MF Su CY Chen HL Chen CM Lactoferrin as a natural regimen of selective decontamination of the digestive tract: Recombinant porcine lactoferrin expressed in the milk of transgenic mice protects neonates from pathogen challenges in the gastrointestinal tract J Infect Dis 2009 199 590 8 10.1086/596212 19125673

[B58] Eckert J Niemann H mRNA expression of leukaemia inhibitory factor (LIF) and its receptor subunits glycoprotein 130 and LIF-receptor-beta in bovine embryos derived in vitro or in vivo Mol Hum Reprod 1998 4 957 65 10.1093/molehr/4.10.957 9809677

[B59] Mukherjee P Cao TV Winter SL Alexandrow MG Mammalian MCM loading in late-G_1 _coincides with Rb hyperphosphorylation and the transition to post-transcriptional control of progression into S-phase PLoS ONE 2009 4 e5462 10.1371/journal.pone.0005462 19421323 PMC2674209

